# Filling the Spring Gap in Southern Australia: Seasonal Activity of Four Dung Beetle Species Selected to Be Imported from Morocco

**DOI:** 10.3390/insects16050538

**Published:** 2025-05-20

**Authors:** Hasnae Hajji, Abdellatif Janati-Idrissi, Alberto Zamprogna, José Serin, Jean-Pierre Lumaret, Nassera Kadiri, Saleta Pérez Vila, Patrick V. Gleeson, Jane Wright, Valérie Caron

**Affiliations:** 1 Laboratoire de Biotechnologie, Conservation et Valorisation des Ressources Naturelles, Faculté des Sciences de Dhar El Mehraz, Université Sidi Mohamed Ben Abdellah, B.P. 1796 Fès-Atlas, Fez 30000, Morocco; hajjihasnae123@gmail.com (H.H.); nermine2002@yahoo.fr (A.J.-I.); 2 CSIRO European Laboratory, 830 Avenue du Campus Agropolis, 34980 Montferrier-sur-Lez, France; alberto.zamprogna@csiro.au (A.Z.); jose.serin@csiro.au (J.S.); jean-pierre.lumaret@univ-montp3.fr (J.-P.L.); 3 Laboratoire de Zoogéographie, Université de Montpellier Paul Valéry, Route de Mende, 34199 Montpellier, France; 4 CEFE, EPHE, IRD, Université de Montpellier Paul Valéry, Route de Mende, 34199 Montpellier, France; nassera.kadiri@univ-montp3.fr; 5 CSIRO Health and Biosecurity, Black Mountain, 2-40 Clunies Ross St., Canberra, ACT 2601, Australia; saleta.perezvila@csiro.au (S.P.V.); patrick.gleeson@csiro.au (P.V.G.); ejanewright@gmail.com (J.W.)

**Keywords:** Scarabaeidae, biological control, species introduction, *Onthophagus*, *Gymnopleurus*

## Abstract

Dung beetles are important ecosystem engineers, as they utilize the excrement produced by animals. For nearly 60 years, several species of dung beetle have been introduced to Australia to help mitigate the problems caused by the accumulation of livestock dung. The twenty-three successfully established species directly contribute to reducing the environmental impacts from dung accumulation, providing improvements to soil health, pasture productivity and pest fly reduction. Despite this success, there are still geographical and seasonal gaps in dung beetle activity, causing dung to remain on the soil surface. The continued importation of new dung beetle species is warranted to fill these gaps. One of the significant remaining gaps is during spring in southern Australia. Four spring-active dung species from Morocco were selected for a new importation program (2018–2022): *Euonthophagus crocatus*, *Onthophagus vacca*, *Onthophagus marginalis* subsp. *andalusicus* and *Gymnopleurus sturmi*. These species were surveyed at four sites in Morocco on an altitudinal gradient to assess their seasonal activity. The four species were found at all sites during spring, but in varying abundances, with different species dominating different sites. This is most likely due to differences in local conditions such as soil type. Seasonal activity varied depending on elevation. *Gymnopleurus sturmi* was found to be active later in the season and should be considered as a summer species. The four species selected will be, if they establish, a useful addition to the already introduced and established dung beetle fauna in Australia.

## Simple Summary

Several dung beetles have been introduced to Australia to help mitigate the problems caused by the accumulation of livestock dung. While dung beetles have been beneficial, there are still geographical and seasonal gaps in dung beetle activity, causing dung to remain on the soil surface. Four dung species from Morocco were selected for a new importation program (*Euonthophagus crocatus*, *Onthophagus vacca*, *Onthophagus marginalis* subsp. *andalusicus* and *Gymnopleurus sturmi*) to fill the activity gap in spring in southern Australia. These species were surveyed at four sites in Morocco on an altitudinal gradient to assess their seasonal activity. The four species were found at all sites during spring, but in varying abundances, with different species dominating different sites. This is most likely due to differences in local conditions such as soil type. Seasonal activity varied depending on elevation. *Gymnopleurus sturmi* was found to be active later in the season and should be considered as a summer species. The four species selected will be, if they establish, a useful addition to the already introduced and established dung beetle fauna in Australia.

## 1. Introduction

Dung beetles play an important role in the ecosystem, burying dung produced by animals that would otherwise accumulate on the soil surface [1,2]. Australia has close to 500 native dung beetle species. These have co-evolved with native animals, mostly marsupials that produce pellets that are often dry and fibrous [3]. When livestock (cattle, sheep and horses) were introduced to Australia, most native dung beetles were not adapted to use the wet, high-volume dung produced. Dung therefore accumulated on the soil surface, causing pasture fouling, water pollution due to nutrient runoff and provided a habitat for dung-breeding organisms such as the bush fly *Musca vetustissima* Walker, 1849 and the biting buffalo fly *Haematobia exigua* de Meijere, 1903 [4].

Forty-four dung beetle species (Scarabaeidae and Geotrupidae), mostly from Africa and Europe, were introduced and released in Australia since 1968, of which twenty-three have established [4,5,6]. These beetles have provided important ecosystem services such as improving soil and pasture, and reducing pest fly populations [1,7]. However, seasonal and geographical gaps in dung beetle activity remain, allowing dung to accumulate on the soil surface. One of these gaps is early spring in the southern regions of Australia. In fact, the analysis of the distribution of introduced dung beetles has shown that Australia’s tropical and subtropical cattle grazing areas are served by 7–13 species of dung-burying beetles, whereas temperate pastures have fewer than 5 species [4]. Of these, most emerge in late spring with activity peaking during summer; only one is active during the autumn–winter period [4,5]. The net effect is a two-to-three-month gap in dung burial when dung beetles are mostly absent. Therefore, to mitigate the problems caused by dung accumulation, the introduction of new species of dung beetles is warranted.

Selecting new species for biological control purposes can be difficult [8,9]. In the case of dung beetles, several characteristics need to be considered: (1) the target (i.e., the type of dung to be controlled); (2) the natural distribution of the dung beetle to determine climatic suitability to the proposed introduction location; (3) its seasonal activity, as dung beetles have specific peak activity; (4) soil type, as dung beetles usually have preferences for soil types [10,11,12,13,14]. Soil varies across the landscape; targeting species with different soil preferences would be an advantage. Furthermore, dung beetle abundance is important to consider, avoiding any impact of removing several thousand individuals on local populations. Consequently, the species conservation status needs to be assessed, as some species are now threatened [15,16]. If a species with the appropriate characteristics described above was successfully introduced elsewhere, it should be prioritized.

Morocco was chosen as the country where dung beetles would be sourced. This country has a wide range of climates caused by its proximity to the sea and various altitudes, from hot desertic to Mediterranean and continental climates [17]. This, coupled with a widespread livestock herding system, produces a diverse and abundant dung beetle fauna [16]. Based on the criteria above and using information found in the literature, four common Moroccan species were selected: *Euonthophagus crocatus* (Mulsant & Godart, 1870), *Onthophagus vacca* (Linnaeus, 1767), *Onthophagus marginalis* subsp. *andalusicus* (Waltl, 1835) and *Gymnopleurus sturmi* (MacLeay, 1821). The first three are paracoprid or tunneller species. Paracoprids excavate tunnels directly under the dung pad, removing dung from the pad to pack into the tunnels to fashion into broods. *Onthophagus* species tend to produce individual elongated tear-shaped broods in which the female lays a single egg. In contrast, *G. sturmi* is a telecoprid or roller species. Telecoprids carve out balls from the dung pad and roll them some distance away before burying them in the soil, where the female deposits a single egg. Dung beetles from different guilds occur in dung beetle communities and can vary spatially and seasonally, e.g., Refs. [18,19], hence, including tunnellers and rollers could be an asset.

*Onthophagus vacca* and *O. m. andalusicus* were first imported into Australia in the 1980s. Australia has strict quarantine regulations, and beetle rearing must first take place in an approved quarantine containment facility before being released into the environment. At this time, quarantine rearing of *O. vacca* and *O. m. andalusicus* proved to be difficult, with only *O. vacca* being released from quarantine. A total of 710 of *O. vacca* were released into the field at two sites, but these failed to establish, most likely due to the small number released [4]. *Onthophagus vacca* was imported again between 2011 and 2014 from France [6]. To increase genetic diversity and potential for drought resistance in this species, further importations from Morocco were suggested by dung beetle experts [20].

Previous dung beetle introductions have focused on cattle dung burial [4]. However, unburied sheep dung also fouls pastures [21], and bush flies will breed in sheep dung [22]. The national herd of sheep stands at about 70 million animals [23]. The four selected species can utilize both cow and sheep dung [24], so they will likely provide control for both sheep and cattle farming areas. This will be of benefit to southern Australia, given the concentration of sheep farming throughout the region. These beetles only consume dung, and therefore they pose no risk to the flora and fauna. Furthermore, they should not overlap much with native dung beetle species, which are not found abundantly in livestock dung [3,7].

This study aims to understand the seasonal abundance of the four selected species in their native range, specifically Morocco, where the beetles destined for Australia will be sourced. While there have been studies on dung beetles in Morocco, few have been focused on these species specifically, except recently [25,26,27,28]. Understanding their seasonal activity will help predict when the beetles will be active in Australia and where they should be released into the environment.

## 2. Materials and Methods

### 2.1. Description of Studied Species

#### 2.1.1. *Euonthophagus crocatus*

**Figure 1:**
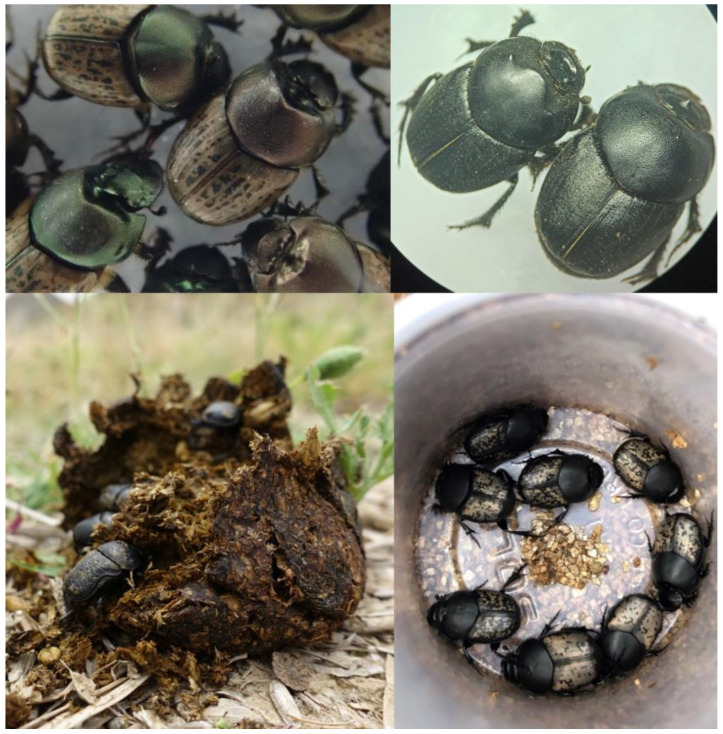
Four dung beetle species in this study. Clockwise from top left: *Onthophagus vacca*, *Euonthophagus crocatus*, *Onthophagus marginalis* subsp. *ndalusicus* and *Gymnopleurus sturmi*.

*Euonthophagus crocatus* (Figure 1) is a medium-sized dung beetle 6–12 mm long [29,30]. It is active during winter–spring [31] or spring–summer [32], depending on the region [24]. *Euonthophagus crocatus* is found in the southern parts of the Mediterranean basin, specifically northern Africa (Morocco, Algeria, Tunisia and Libya). It is not found in the Saharan regions. There have been reports of this species in southern Italy (Sicily and Calabria) and southern Spain, but these remain unconfirmed [32]. *Euonthophagus crocatus* is abundant in Morocco [24,32,33]. *Euonthophagus crocatus* prefers cattle and sheep dung, but will also eat horse dung, and occasionally goat dung or human feces [24,33].

*Euonthophagus crocatus* prefers open habitats and humid pastures but can also be found in forested areas. This species has been collected from 124 m up to 2050 m in altitude [24,34]. *Euonthophagus crocatus* has been collected by several studies using traps with cow dung as bait [33]. However, in the area where these studies were conducted, sheep (*Ovis aries*) were the most abundant animal [24].

#### 2.1.2. *Onthophagus marginalis* subsp. *andalusicus*

*Onthophagus andalusicus* was reclassified as a subspecies of *Onthophagus marginalis* by some authors [35]. *Onthophagus marginalis marginalis* has an Asian distribution (Eastern Europe, from Russia to China and the Korean peninsula), while *O. marginalis andalusicus* has a more western distribution [36]. *Onthophagus m. andalusicus* (Figure 1) is 6–12 mm long [29,30]. It is mostly distributed throughout the western area of the Mediterranean Basin. It is found in northern Africa (Morocco, Algeria and Tunisia), except in the Saharan regions, and in southwestern Europe (Portugal, Spain, Italy and Malta) [37]. This species is abundant in Morocco, Spain and northern Tunisia [37]. *Onthophagus m. andalusicus* prefers cattle and sheep dung [18,38] and is active during spring and early summer [18,38]. It is a common species in Morocco, where it can dominate dung beetle communities in spring from February to June but can be found as late as August [39]. At some sites, it was so dominant that it represented more than 70% of the dung beetle number present and 65.7% of the biomass [39].

#### 2.1.3. *Onthophagus vacca*

*Onthophagus vacca* (Figure 1) is slightly larger than the other two tunnellers in this study, being 7–13 mm long [29,30]. *Onthophagus vacca* is a common species in Europe and is found in Morocco, Asia Minor (Turkey), Syria and Iran [29,30]. Until recently, it was thought that there was a wide range of color morphs in this species, from very light (or clear) elytra to very dark. *Onthophagus medius* (Kugelann, 1792) (the dark form) is indeed a separate species from *O. vacca*, based on morphology as well as molecular phylogeny [40,41]. The distributions of the two species overlap widely, but *O. medius* is a more cold-tolerant species, occurring in England, Belgium, northern Germany and northern Poland, where *O. vacca* does not occur, but is absent in southern Spain, Morocco and Sardinia, where *O. vacca* does occur. In the overlap zone, *O. vacca* is more likely to be found at low elevations and *O. medius* at higher elevations [40]; however, the two species can coexist at the same site, sometimes in the same dung pad.

*Onthophagus vacca* is active in spring and summer. Adults emerge in early spring (March–April) and feed on dung for several weeks until they are ready to reproduce [10]. Adult offspring emerge in late summer, feed for a period and then enter an obligatory winter diapause until the following spring [42]. *Onthophagus vacca* is mostly attracted to fresh cattle and sheep dung in open pastures [10]. A study by Lumaret and Kirk [10] showed that *O. vacca* has a very strong preference for open, non-shaded sites.

#### 2.1.4. *Gymnopleurus sturmi*

*Gymnopleurus sturmi* (Figure 1) is a medium-sized beetle 10–15 mm long [29,30]. This species is widely distributed throughout the Mediterranean Basin, and it has been recorded in Portugal, Spain, the Balearic Islands, France, Corsica, Italy, Sardinia, Sicily, Croatia, the Syrian Arab Republic, Iraq, Lebanon, Jordan, occupied Palestinian Territory, Israel, Saudi Arabia, Egypt, Tunisia, Algeria and Morocco [43]. A decline in presence and abundance has been observed in France, Italy, Spain and Portugal during the past 30 years, hence its inclusion on the IUCN Red List as a Near Threatened species. This decline is most likely due to the change in livestock management and the reduction of habitats. Nevertheless, the species is observed to be very abundant in northern African dung beetle assemblages [25,38,43,44].

*Gymnopleurus sturmi* prefers sheep dung, but also eats cattle dung, horse dung and human and dog feces [38,43,45]. Preferred sites include grasslands, shrublands, sandy beaches and agricultural sites, preferably with clay or sandy soil [10,43]. In France, *G. sturmi* is active from spring to early autumn, breeding during late spring and early summer [46]. In Morocco, activity occurs from late winter to late summer, with some activity in mid-autumn. Breeding occurs during mid-to-late spring [47].

### 2.2. Site Descriptions

**Figure 2:**
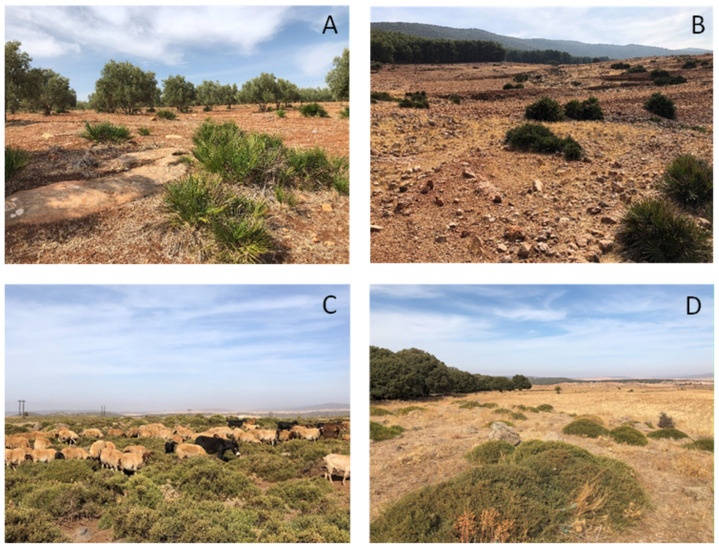
Photos of the four sites taken in October 2019. (**A**) Fez-Sais, (**B**) Imouzzer, (**C**) Ifrane 1 with mixed herd, (**D**) Ifrane 2.

Four sites were sampled in 2018–2019 in Morocco. Sites were chosen based on previous studies indicating the presence of all four target species, e.g., Refs. [33,38,39]. Elevation varied between sites and ranged from 600 m to 1631 m (Table 1). Sites also differed from each other based on their environmental characteristics (Table 1, Figure 2). As practiced in Morocco, shepherds move through the landscape daily with their mixed herds of animals, predominantly consisting of sheep but may also include goats, donkeys and some cattle (Figure 2C). Therefore, different herds would pass through the sites several times a day.

### 2.3. Sampling

Forty-two surveys were carried out at Ifrane 1 and Ifrane 2 between April 2018 and August 2019, and thirty-six surveys were carried out between September 2018 and August 2019 at the Fez-Sais (=Ain Cheggag) and Imouzzer sites. The number of surveys varied according to the season, between one and five per month, depending on dung beetle abundance at that time of the year and the seasonal activity of the species being studied. For example, very few dung beetles were active in the winter months, especially in Ifrane, due to snow cover. Therefore, fewer surveys were conducted during the winter months.

**Figure 3:**
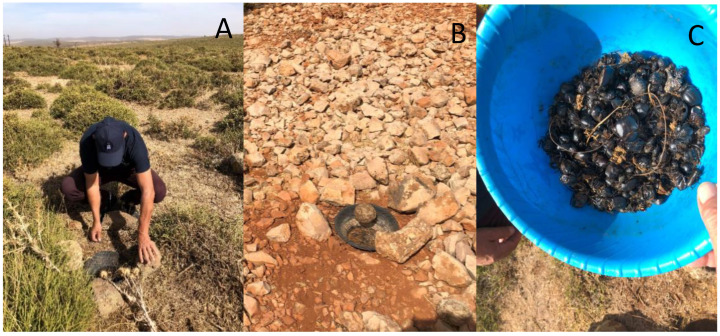
Dung beetle trap (**A**) set up at Ifrane 1, (**B**) trap to be reset at Imouzzer, (**C**) dead dung beetles in trap.

Three CSR-type baited traps [49] were set up 50 m apart at each of the study sites [50]. The traps consisted of a round plastic dish 30 cm in diameter and 15 cm deep. The traps were buried flush with the soil level. Each dish was covered with a piece of steel mesh 40 × 40 cm (1 cm^2^ aperture). Approximately 250 g of fresh cattle dung was placed on top of the mesh to act a bait. The dishes were filled with soapy water mixed with alcohol (30% volume) or, more often, a few drops of formalin (Figure 3). Traps were removed 7 days after installation. All beetles were preserved in 95% ethanol, sorted and identified to species.

### 2.4. Analyses

Monthly meteorological data (maximum, minimum and mean temperature, and mean precipitation) were obtained from the Watershed Agency of Sebou (Agence du Bassin Hydraulique de Sebou) for each site. As Ifrane 1 and 2 are geographically close, the same data were used for both sites.

The proportion of a species present at a site at a given time was calculated by dividing the number collected at a sampling event for that species by the total number of individuals collected for all species combined [19]. For some surveys, some of the traps disappeared or were destroyed by humans or animals. For this reason, the average number of beetles per trap for a given month was used to compare dung beetle abundance through time and between sites and was plotted.

To assess if there were differences in the trapped rate between sites, pairwise comparisons were performed separately for each species in JMP (version 18.2.0, SAS Institute Inc., Cary, NC, USA). Due to the large number of zeros in the dataset, the data were transformed using log natural (x + 1) prior to the analysis.

## 3. Results

**Figure 4:**
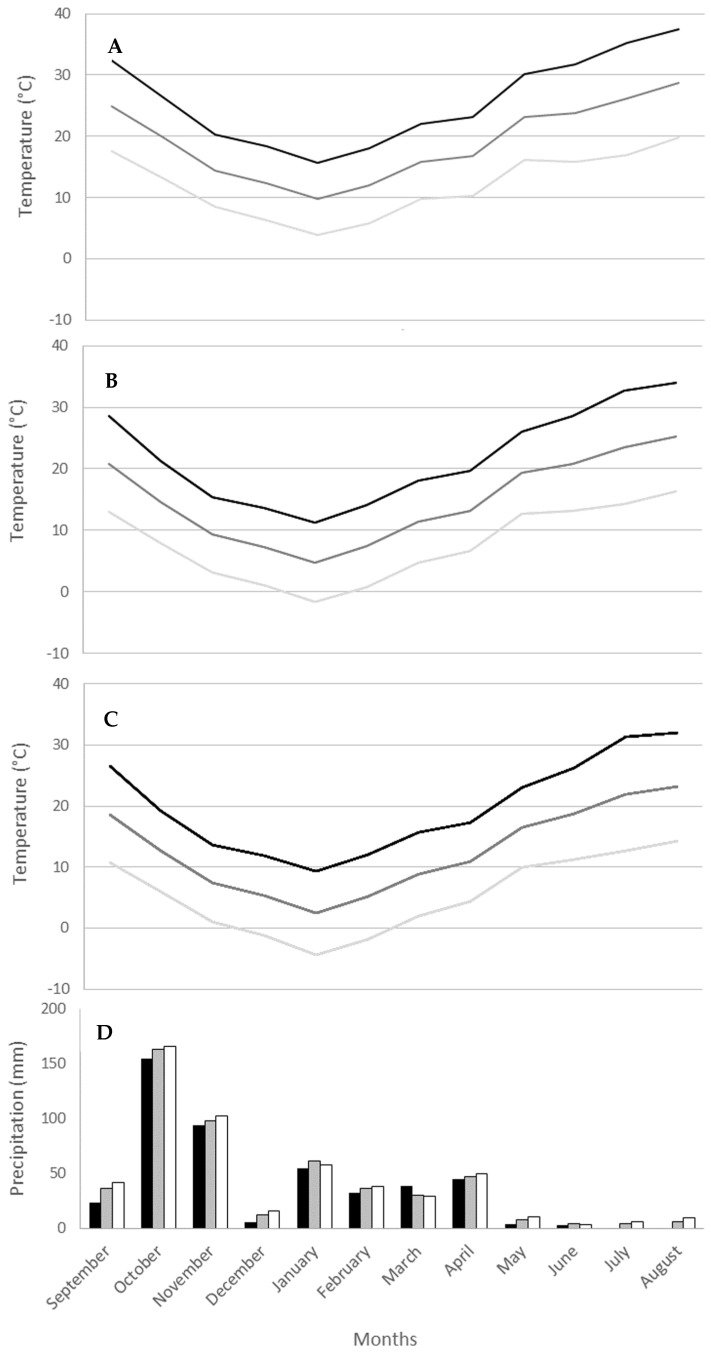
Monthly maximum temperature (black), monthly mean temperature (dark gray), monthly minimum temperature (light gray) between September 2018 and August 2019 for sampling sites for (**A**) Fez-Sais, (**B**) Imouzzer, (**C**) Ifrane; and (**D**) mean monthly precipitation (mm) for sampling sites Fez-Sais (black), Imouzzer (gray), Ifrane 1 and 2 (white).

Throughout the sampling period in 2018–2019, the monthly average temperatures (minimum, maximum and mean) showed identical patterns for all sites (Figure 4). As expected, there was a decrease in average annual temperature with increasing altitude, from Fez-Sais, the lowest, to Ifrane, the highest site (Figure 4). The average annual temperature was approximately 4 degrees higher in Fez-Sais (18.95 °C) than in Imouzzer (14.80 °C) and was also 2 degrees warmer on average than in Ifrane (12.63 °C). The average precipitation was higher in autumn through October and November at all sites, with an altitudinal gradient from Fez-Sais (458.7 mm/year) to Imouzzer (504.7 mm) and Ifrane (527.7 mm) (Figure 4).

**Figure 5:**
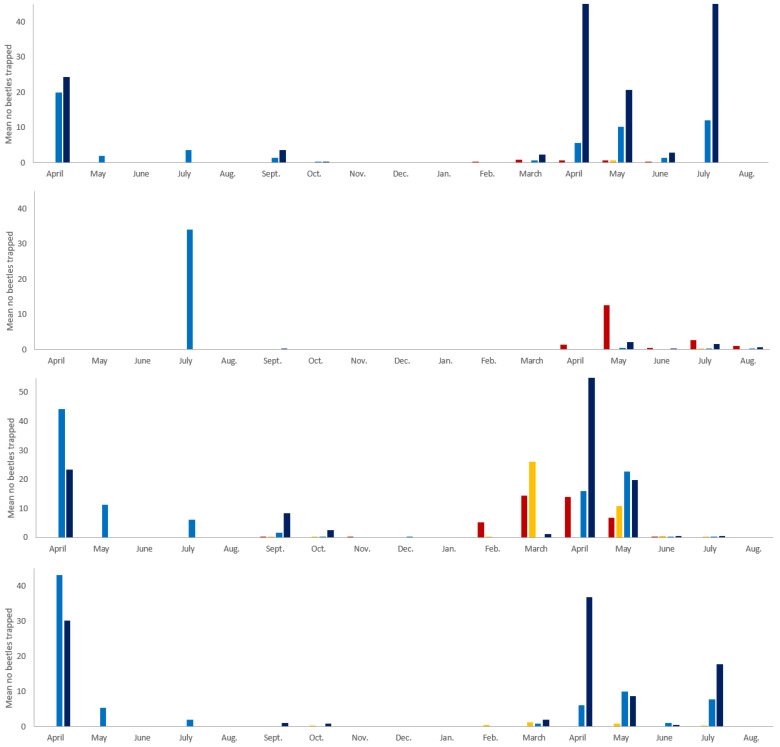
Mean number of beetles trapped monthly at four sites in Morocco between April 2018 and August 2019 (from **top** to **bottom**: *Euonthophagus crocatus*, *Onthophagus vacca*, *Onthophagus marginalis* subsp. *andalusicus*, *Gymnopleurus sturmi*, red: Fez-Sais, yellow: Imouzzer, light blue: Ifrane 1, dark blue: Ifrane 2). No sampling occurred at Fez-Sais and Imouzzer between April and September 2018.

A total of 51 species were found during this study, including 27 Scarabaeinae species, 21 Aphodiinae species and 3 Geotrupidae species [19]. Imouzzer had the lowest diversity with 35 species, followed by Fez-Sais with 39 species. Diversity was slightly higher at both Ifrane sites, where 42 and 43 species, respectively, were captured [19]. The 4 target species were found at all sites, but with differing seasonal abundances (Figure 5). There were more beetles collected in Ifrane 1 and Ifrane 2 than in Imouzzer and Fez-Sais, with the lowest average numbers collected in Fez-Sais.

*Euonthophagus crocatus* appeared first during the season, being present in February at Fez-Sais (Figure 5). It was caught from March to June in Imouzzer and from April in Ifrane 1 and Ifrane 2. Interestingly, it was present until May at all sites and reappeared in July in Ifrane 1. It dominated Imouzzer for much of the spring and represented up to 81% of individuals trapped between March and May. While the percentages were lower at the other sites, up to 45% of trapped individuals between March and May in Fez-Sais belonged to this species, and up to 44% in May in Ifrane 1. This species was rarer in Ifrane 2 but still made up 28% of the total individuals caught in May.

*Onthophagus vacca* and *O. m. andalusicus* followed the same patterns across all sites (Figure 5). They were rare in the lower altitude sites (Fez-Sais and Imouzzer) for most of the year, except for two trapping events in June, where they represented close to 50% of beetles caught in Fez-Sais; however, the total abundance of dung beetles captured was relatively low. At high altitude sites (Ifrane 1 and Ifrane 2), *O. vacca* and *O. m. andalusicus* were abundant from April to May. *Onthophagus vacca* made up to 33% and 27% of the dung beetle community caught at Ifrane 1 and Ifrane 2, respectively, over those two months. At the same sites over the same period, *O. m. andalusicus* represented 17% and 42% of beetles caught. Abundance of both species declined considerably through June; however, both species reappeared in July at the two Ifrane sites (Figure 5).

*Gymnopleurus sturmi* was the latest species to appear and was active at all sites from May (Figure 5). It was most common in Fez-Sais, where it occurred from May to August and made up between 22% and 100% of all dung beetles caught. It was rare in Imouzzer, only being trapped in low numbers on three occasions. *Gymnopleurus sturmi* was more abundant in July and August at the Ifrane sites. In 2019, it made up 22% and 36% of all dung beetles caught for those two months, respectively.

The average number of dung beetles caught in Ifrane varied between the two sampling years. In 2018, there were more beetles trapped overall than at a similar time in 2019 (Figure 5).

When comparing trapped numbers between sites, Ifrane 1 and Ifrane 2 had R^2^ above 0.7 for all four species. The other pairwise comparisons did not show any relationship (Table 2).

## 4. Discussion

There is a clear gap in dung beetle activity in spring in southern Australia [4]. During spring, there are very few introduced dung beetles active, causing dung to accumulate on the soil surface. Therefore, there has been a renewed interest in further dung beetle importation programs to fill this lack of dung beetle activity. Four Moroccan species have been selected and assessed as part of the most recent importation program [20]. The four selected species were present at all four study sites throughout the Moroccan spring and summer. Although seasonal activity varied between the species, the selected species were often the dominant species occurring during spring [19].

*Euonthophagus crocatus* was the first to appear, followed by *O. vacca* and *O. m. andalusicus*. Although *G. sturmi* appeared concurrently, it became dominant as the season progressed, with abundance peaking in late spring and summer. Interestingly, the seasonal activity results from this study were slightly different from previous studies. For example, *E. crocatus* has been defined as a winter and spring species, with a maximum abundance from mid-spring to early summer [32]. In this study, the earliest that *E. crocatus* was collected was in late winter (February) at Fez-Sais, the lowest altitude site that experiences warmer temperatures compared with the other three sites, with a mean temperature of 11.95 °C in February, which is equivalent to the mid-April mean temperature in Ifrane (Figure 4).

Janati-Idrissi [33] found that *G. sturmi* is active from late winter to late summer (February–August) in Morocco, with a maximum abundance in mid to late spring (April–May). This study suggests a later seasonal activity for *G. sturmi*, peaking in May and July, as found in Fez-Sais, which was the site where it was most abundant. At the cooler, higher altitude sites, they were a rare occurrence, but were more abundant in summer, where they can withstand high temperatures at ground level [25]. The results suggest that *G. sturmi* cannot be defined as being primarily a spring species, as first thought, like the other three species. Nevertheless, the activity of the four species selected matches the targeted spring seasonal activity gap in southern Australia. The dominance of these species observed in the four sites is promising for Australia.

Seasonal activity and abundance for each species also varied depending on the site. For example, the lowest altitude sites (Fez-Sais and Imouzzer) had a higher proportion of *E. crocatus*, while Ifrane 1 and Ifrane 2 had more of *O. vacca* and *O. m. andalusicus*. Interestingly, there were differences between the two sites in Ifrane despite being geographically close. However, there was a clear relationship between these two sites, as indicated by the high R^2^ when doing pairwise comparisons for all four species. None of the other comparisons showed any relationship sites at lower altitudes. The beetle number trapped therefore followed the same pattern at the two higher altitude sites, but not at the two other sites.

Dung beetle abundance and activity are influenced by several factors, including temperature, vegetation cover and soil type, e.g., Refs. [12,13,34,38,51,52]. The difference in abundance of the four selected species could be explained by site characteristics. For example, *E. crocatus* prefers open habitats and humid pastures but is sometimes found in forested areas [24,53], while *G. sturmi* prefers open, dry sites, including grasslands, shrublands and agricultural sites [46,54,55], and clay or sandy soil [43,46]. *Onthophagus m. andalusicus* generally prefers wetter habitats in dry, hot areas of the Mediterranean, such as lagoons, salt marshes, coastal areas and humid pastures [31,37,52,56]. However, its presence on hard and rocky soil in Fez-Sais and on dry clay-loam soils in Ifrane shows the plasticity of this species. Its abundance in Ifrane also shows that it can withstand both low winter temperatures (average minimum temperatures ranging from −1.2 to −4.4 °C between December and February, with some periods of snow) and high summer temperatures between April and July.

Population-limiting factors in the natural range of *E. crocatus*, *O. m. andalusicus* and *G. sturmi* include resource availability and competition with other dung beetle species. Dung beetle species occupying the same niche will compete for resources such as dung and space [57]. During late winter and spring, new species, once established in Australia, will have very limited competition from the existing introduced species, as this niche is mostly empty. Of the introduced species that are present during this period, *Bubas bison* activity declines sharply after mid-winter; *Copris hispanus* is active in spring, but its distribution is limited to the region of its original release in western Australia; and *Onitis caffer*, although it is active in winter and spring, is also not widely distributed [4,5].

The same cannot be said about summer. There are already several introduced species active from early summer [4,5], including some very dominant species [58]. *Onthophagus vacca* and *O. m. andalusicus* breed in spring [10]. Once emerged, their adult offspring will feed for several weeks and will only breed in the following spring after their winter dormancy [6]. Therefore, these two species will be in competition with the highly abundant summer beetles before they can breed. Several of the introduced species already co-exist with the four selected species in the native range (i.e., *Euoniticellus fulvus*, *Onthophagus taurus* and *Onitis alexis*) [5,19]. *Onthophagus vacca* and *O. m. andalusicus* should therefore cope with the competition. However, the level of competition experienced may differ in the native and introduced ranges. For example, in some parts of western Australia, *O. taurus* is the dominant species, and thousands of individuals can be trapped within 24 h [1]. The locations for the release of new beetles should consider the abundance of already introduced species outside their breeding season to increase the chance of establishment.

Results from this study indicate that *G. sturmi* is a species that prefers high temperatures. This is supported by Hajji et al. [25], that observed individuals being active when the surface temperature was around 50 °C. Furthermore, while it is active at lower temperatures, it will not breed until higher temperatures are reached (pers. observation). There are no introduced roller species in southern Australia, where the temperatures can be very high during summer. If it succeeds, it will be the first species of this guild that will use livestock dung in these southern regions. *Gymnopleurus sturmi* aggregates in large numbers on dung pads [25], and it is possible that it puts pressure on already introduced species if it establishes and reaches high populations. However, *G. sturmi* was only dominant at one site, and as mentioned above, it would already co-exist with many of the species present in Australia, and an equilibrium would eventually be reached.

This study found a net decrease in *G. sturmi* abundance compared to previous studies. In 1994, Janati-Idrissi et al. [33] collected up to 2300 individuals per trap, compared to an average of 30 per trap in 2019. Fez-Sais is becoming increasingly degraded due to the urbanization of the region, restricting the presence of grazing animals. The degradation of the environment and the reduction of suitable grazable pastures (with a correlating reduction in the number of animals) seem to be the main reasons for the observed decline over 30 years. Land uses, especially urbanization and intense agriculture, are major factors affecting dung beetle density [59].

*Gymnopleurus sturmi* is listed as a Near Threatened species on the IUCN Red List under criterion B2 [16]. Across its range, this species appears to have declined in presence and abundance in Western Europe (Spain, Portugal, France and Italy) over the past 30 years but remains very abundant in northern Africa [43]. A recent study in a site 80 km north of the city of Fez, Morocco, showed that this species remains abundant, with populations in good condition [25]. However, the decline observed in this study is worrying, and further work to determine if the decline of this species is occurring locally or globally is warranted.

In biological control, the number of species introduced is an important factor to be considered, as there is a risk associated with each new introduction [60]. In this case, introducing several species is warranted. Importing a suite of species instead of one would be beneficial in this case, as there are clear differences in seasonal activity and abundance between sites, even closely located sites. Dung beetles have specific preferences for certain habitat variables, such as soil and dung types [10,61]. Therefore, introducing several species would increase the chance of having beetles suited to different conditions and geographical areas. All four species studied here, *E. crocatus*, *O. m. andalusicus*, *O. vacca* and *G. sturmi*, are found in assemblages of other pasture-dwelling dung beetle species in their native range [34,38,39,44,62]. These species clearly inhabit the same niche and do not appear to severely limit each other’s activity. The risk of these species having a negative impact on already introduced dung beetle species is very low, as their activity peaks occur at different times. Furthermore, the success of any new introduction is not guaranteed. Out of the 44 species introduced to Australia so far, 23 have been considered as established [4], a 52% success rate. Introducing 4 species may increase the chance of at least some species establishing successfully.

## Figures and Tables

**Table 1:** Site description and location.

Site	Location	Altitude	Site Characteristics	Main Grazers
Fez-Sais (Ain Cheggag)	33°54′14″ N–4°59′55″ W	609 m	Open, mostly flat environment. Herbaceous vegetation, with *Asphodelus ramosus* F. and sparse trees (*Pistacia terebinthus* L.), close to olive orchards. Karstic formation of lacustrine origin, composed mainly of massive limestone and travertine associated with conglomerates and marl, resulting in a hard and rocky soil. Semi-arid bioclimatic zone with temperate winters. Hot summer Mediterranean climate [17].	Sheep Goats A few cattle
Imouzzer Kandar	33°47′53″ N–4°59′21″ W	898 m	Open, overgrazed scrubland environment. Herbaceous vegetation with a few patches of *Chamaerops humilis* L. (palm) (locally called doum) and more closed areas of holm oak (*Quercus ilex* L.) coppice. Gentle slope. Rocky area interspersed in clear patches of soil/vegetation. The substratum consists of superficial red clay soil, with outcrops of limestone blocks. The climate is classified as Csa according to the Köppen–Geiger classification, with hot, dry summers and mild, wet winters [48].	Sheep Donkeys
Ifrane 1	33°32′42″ N–5°09′56″ W	1631 m	Open, flat roadside site. Herbaceous vegetation with dense broom cover (*Genista quadriflora* Munby), numerous clumps of *Chamaerops humilis*, indicating intensive grazing by sheep. Rocky, with a superficial red soil made of silty clay. Many volcanic stones on the surface. Humid bioclimatic zone with cold winter and warm summer, Mediterranean climate [17].	Sheep Goats Donkeys
Ifrane 2	33°33′03″ N–5°10′02″ W	1613 m	Open environment, grasslands, with few brooms near forested areas (holm oak) and cropped fields. Superficial red soil made of silty clay. Volcanic stones on the surface. Humid bioclimatic zone with cold winter and warm summer, Mediterranean climate [17].	Sheep Cattle Horses Donkeys

**Table 2:** Pairwise comparisons of the trapped beetle number between sites; *p* value and equation were provided when R^2^ was above 0.7.

*Euonthophagus crocatus*				
	**Fez**	**Imouzzer**	**Ifrane 1**	**Ifrane 2**
**Fez**	-			
**Imouzzer**	R^2^ = 0.21	-		
**Ifrane 1**	R^2^ = 0.22	R^2^ = 0.28	-	
**Ifrane 2**	R^2^ = 0.19	R^2^ = 0.35	R^2^ = 0.78 *p* < 0.001 y = 0.200 + 0.903x	-
*Onthophagus vacca*				
	**Fez**	**Imouzzer**	**Ifrane 1**	**Ifrane 2**
**Fez**	-			
**Imouzzer**	R^2^ = 0.03	-		
**Ifrane 1**	R^2^ = 0.001	R^2^ = 0.18	-	
**Ifrane 2**	R^2^ = 0.004	R^2^ = 0.30	R^2^ = 0.67 *p* < 0.001 y = 0.236 + 0.882x	-
*Onthophagus m. andalusicus*				
	**Fez**	**Imouzzer**	**Ifrane 1**	**Ifrane 2**
**Fez**	-			
**Imouzzer**	R^2^ = 0.01	-		
**Ifrane 1**	R^2^ = 0.01	R^2^ = 0.12	-	
**Ifrane 2**	R^2^ = 0.07	R^2^ = 0.31	R^2^ = 0.75 *p* < 0.001 y = 0.240 + 1.125x	-
*Gymnopleurus sturmi*				
	**Fez**	**Imouzzer**	**Ifrane 1**	**Ifrane 2**
**Fez**	-			
**Imouzzer**	R^2^ = 0.003	-		
**Ifrane 1**	R^2^ = 0.10	R^2^ = 0.02	-	
**Ifrane 2**	R^2^ = 0.04	R^2^ = 0.003	R^2^ = 0.82 *p* < 0.001 y = 0.075 + 2.013x	-

## Data Availability

The raw data supporting the conclusions of this article will be made available by the authors on request.
